# Surgical management of intraocular lens dislocation in highly myopic eyes

**DOI:** 10.3389/fmed.2026.1922084

**Published:** 2026-10-05

**Authors:** Shiyu Tang, Chenxi Li, Zihan Xie, Fan Yang, Yuye Zhang, Wenting Cai, Tianyu Zheng

**Affiliations:** 1 Department of Ophthalmology, Shanghai Tenth People’s Hospital, Shanghai, China; 2 Visual Rehabilitation Center, Tongji University School of Medicine, Shanghai, China; 3 Tongji Eye Institute, Tongji University School of Medicine, Shanghai, China; 4 Department of Ophthalmology, Eye & ENT Hospital, Fudan University, Shanghai, China

**Keywords:** intraocular lens dislocation, intraocular lesns positional stability, IOL repositioning surgery, surgical safety, WF-OCTA

## Abstract

**Objective:**

To evaluate the postoperative visual function, intraocular lens (IOL) positional stability and ocular safety of surgical strategies tailored to capsular support status for the treatment of IOL dislocation in patients with high myopia, and to explore the potential impact of such complex surgeries on macular microcirculation.

**Methods:**

This single-center retrospective included 28 patients (28 eyes) with high myopia and IOL dislocation who underwent surgical treatment from January 2024 to August 2025. Surgical approaches were selected based on preoperative anatomical conditions and capsular status, including IOL explantation without secondary IOL implantation in 10 eyes, in-the-bag IOL repositioning in 6 eyes, and sutured scleral fixation of a posterior chamber IOL in 12 eyes. The primary outcome measures were uncorrected distance visual acuity (UDVA), corrected distance visual acuity (CDVA), and IOL tilt and decentration before surgery and at 6 months postoperatively. Safety measures included intraocular pressure (IOP), corneal endothelial cell density (ECD), postoperative complications. Postoperative retinal microcirculation parameters assessed by WF-OCTA were included as exploratory outcomes. Whereas the IOL-position comparison included only the 18 operated eyes retaining an IOL and their corresponding 18 matched control eyes. Before quantitative analysis, all OCTA images were reviewed for image quality.

**Results:**

Compared with preoperative values, UDVA (0.59 ± 0.46 logMAR, *P* = 0.005) and CDVA (0.38 ± 0.59 logMAR, *P* = 0.031) were significantly improved at 6 months postoperatively. Postoperative IOL position was evaluated in the 18 eyes retaining an IOL; 3 (16.7%) exceeded the 6° tilt threshold and 2 (11.1%) exceeded the 0.6 mm decentration threshold. The postoperative 2D tilt of IOL was 4.51 ± 2.17°, 2D decentration was 0.45 ± 0.33 mm, 3D tilt was 4.41 ± 2.09°, and 3D decentration was 0.44 ± 0.34 mm. Corneal ECD decreased from 2672.72 ± 312.26 cells/mm^2^ preoperatively to 2356.61 ± 420.15 cells/mm^2^ postoperatively (*P* < 0.001), yet no corneal decompensation occurred. IOP remained stable during the follow-up period, and the incidence of complications was low. Regional WF-OCTA parameters varied between the operated and matched control eyes, without a consistent pattern across macular regions.

**Conclusion:**

In patients with high myopia and IOL dislocation, different surgical approaches can achieve stable intraocular optical reconstruction and improve mid-to-long-term visual function under the corresponding anatomical conditions. Although the magnitude of visual acuity improvement is limited in this population due to high myopia- related high-risk fundus factors.

## 1 Introduction

Intraocular lens (IOL) dislocation is one of the severe complications after cataract surgery, often leading to decreased visual acuity, monocular diplopia, glare/halos and refractive changes (1, 2). Improper management may further induce secondary glaucoma (38.7%), corneal endothelial damage (8.6%) and irreversible visual impairment. With the aging of the population and the continuous increase in the volume of cataract surgeries, the clinical demand for the management of IOL dislocation is gradually rising (3–5). Meanwhile, the population of patients with high myopia is expanding, and this group is the core high-risk population for late in-the-bag IOL dislocation- literature data show that the proportion of high myopia among patients with IOL dislocation can reach 10%–40% (1, 6). How to ensure safety while achieving stable optical reconstruction and visual recovery in patients with high myopia has become an important concern in the field of ophthalmology.

**Figure 1:**
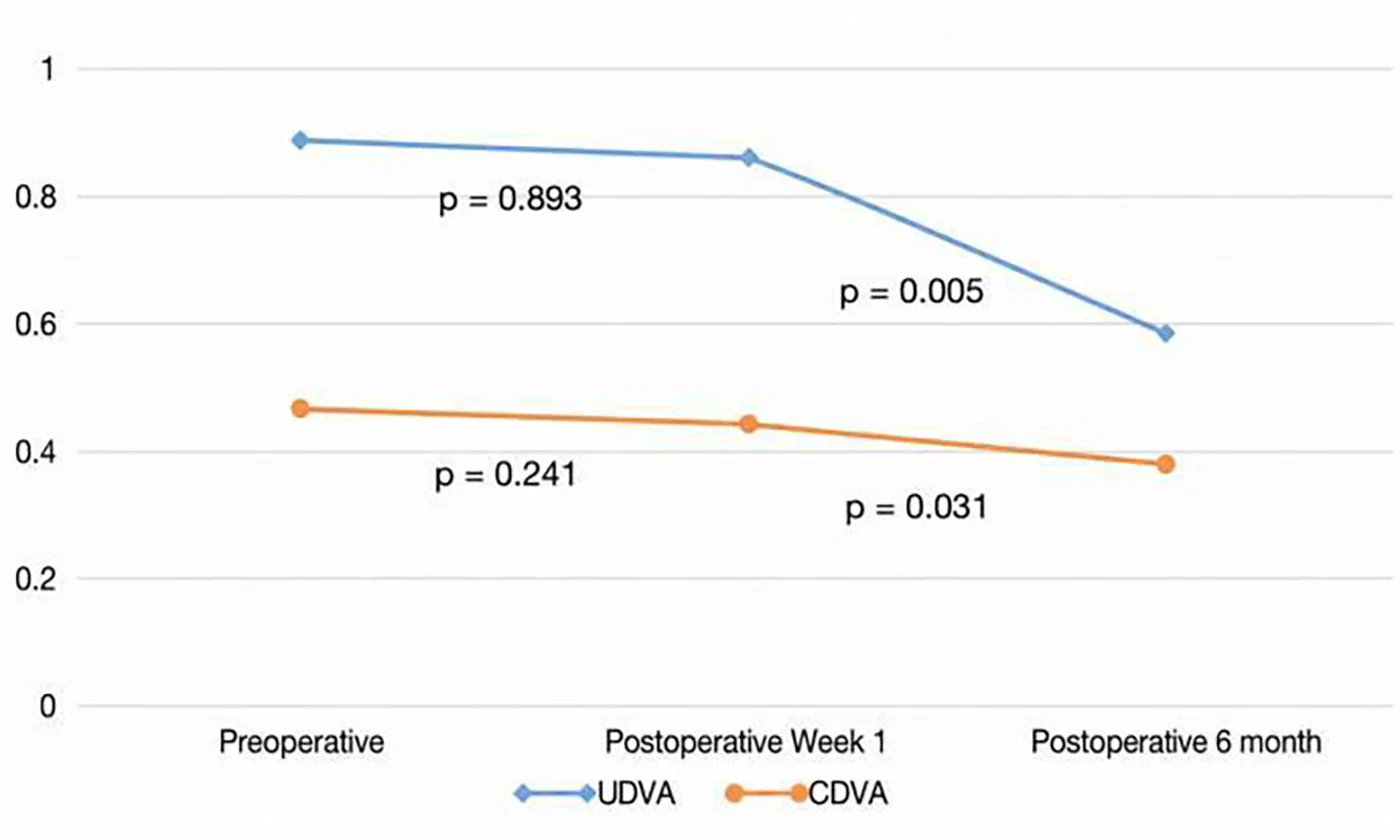
Comparison of preoperative and postoperative CDVA and UDVA. CDVA, corrected distance visual acuity, UD VA, uncorrecteddistance visual acuity.

Current surgical treatment strategies for IOL dislocation depend on the type and degree of dislocation, the integrity ofthe lens-capsule complex, the presence ofvitreous traction, and the patient's fundus status (7). The surgical management of IOL dislocation is mainly determined by the integrity of the lens-capsule complex and its residual supporting capacity. When the capsular structure is intact and can provide stable support, in-the-bag IOL repositioning is the first choice, and capsular tension ring is combined if necessary to enhance long-term stability. If capsular support is insufficient or stable repositioning is difficult to maintain, suture or sutureless scleral fixation can be selected. For patients with high myopia who plan to reserve a refractive status of approximately −3.00 D postoperatively, IOL explantation without secondary IOL implantation can be performed (7, 8). In clinical practice, especially in cases with complex anatomical conditions, the surgical plan needs to be selected according to ocular conditions, and combined anterior or posterior vitrectomy is often required to relieve vitreous traction and reduce perioperative risks (9). Therefore, the management of IOL dislocation in high myopic eyes relies on surgical decisions based on different ocular anatomical conditions and capsular status, and its efficacy and safety still need systematic evaluation.

Notably, patients with high myopia are often complicated with severe fundus structural lesions, such as chorioretinal atrophy, macular-related diseases, and retinal breaks (10, 11). For these patients, even if anatomical reduction of the intraocular optical system is achieved, postoperative visual function recovery may still be affected by the integrity of macular structure, the functional reserve of the retinal neurovascular unit, and previous fundus lesions (12, 13). Therefore, evaluating surgical outcomes solely based on traditional indicators such as visual acuity, refractive status or IOP may not fully reflect the real benefits and potential risks ofthis population.

Wide-field optical coherence tomography angiography (WF-OCTA) is a non-invasive, repeatable imaging tool that can quantitatively assess the vessel density of the superficial capillary plexus (SCP) and deep capillary plexus (DCP) in the macular area,and changes in retinal thickness (14). Different from previous studies on IOL dislocation surgery that mainly focused on visual acuity, refractive status and IOP, OCTA helps to supplement the assessment of postoperative status in complex patients from the perspective of microcirculation and structure (15). Especially in patients with high myopia or a history of previous retinal surgery, changes in macular microcirculation may be more closely related to individual differences in visual function recovery.

Based on the above background, this study aims to evaluate the visual function improvement, IOL positional stability and perioperative safety of surgical approaches selected according to anatomical conditions in patients with high myopia and IOL dislocation. Accordingly, this exploratory retrospective aimed to describe visual outcomes, postoperative IOL position, and ocular safety outcomes after anatomy- guided surgical management of IOL dislocation in highly myopic eyes. Postoperative WF- OCTA parameters and postoperative IOL position were additionally compared, on an exploratory basis, with those of individually matched highly myopic eyes after uncomplicated cataract surgery.

## 2 Methods

### 2.1 Ethical statement

The study was approved by the institutional review board of shanghai tenth people's hospital (SHSY-IEC-6.0/25K281/P01) and conducted in accordance with the Declaration of Helsinki. Written informed consent was obtained from all participants. The study was registered with the Chinese Clinical Trial Registry (ChiCTR2600130426). All research procedures were in accordance with the ethical principles of the Declaration of Helsinki. Before surgery, all participants and their family members were informed of the surgical precautions and potential complications, and written informed consent was obtained from each patient.

### 2.2 Patient selection

This retrospective study enrolled patients with high myopia complicated by IOL dislocation who underwent surgical treatment at the Tenth People's Hospital of Shanghai from January 1, 2024 to August 1, 2025. Inclusion criteria: (1) Diagnosis ofIOL dislocation confirmed by slit-lamp examination, ocular B-ultrasound or anterior segment optical coherence tomography (CASIA-2); (2) A history of high myopia (axial length [AL] ≥ 26 mm or spherical equivalent [SE]≤ -6.0 D); (3) Follow-up time ≥6 months. Exclusion criteria: (1) Preoperative presence of severe ocular diseases that seriously affect visual acuity such as severe corneal opacity; (2) Irreversible visual impairment caused by systemic diseases or ocular trauma after surgery; (3) Incomplete clinical data or loss to follow-up. For the exploratory IOL position comparison, only the 18 operated eyes retaining an IOL and their corresponding 18 matched control eyes were included. Controls were selected from independent patients with high myopia who had undergone uncomplicated routine cataract surgery. Eligibility criteria included an axial length ≥26 mm or a spherical equivalent ≤−6.0 D and uneventful cataract surgery.

All surgeries were performed under local anesthesia by an experienced ophthalmologist (Tianyu Zheng). The surgical approach was selected based on preoperative anatomical conditions, capsular integrity, zonular status, and the type and degree of IOL dislocation: 1)In-the-bag IOL repositioning (6 eyes): Retrobulbar anesthesia with 2% lidocaine was administered, 1.0 mm paracorneal incisions were made at the 3 and 9 o'clock limbal positions, the dislocated/decentered IOL was repositioned into the capsular bag and adjusted to a centered and stable position, and a capsular tension ring was combined if necessary to enhance stability. 2) IOL explantation without secondary IOL implantation (10 eyes): Anesthesia and paracorneal incisions were the same as above, viscoelastic was injected into the anterior chamber to release adhesions between the capsule and IOL before IOL extraction, without implantation ofa new IOL. 3) sutured scleral fixation of a posterior chamber IOL (12 eyes): Following anaesthesia and creation of the paracorneal incisions, adhesions were released and the original IOL was extracted. A 6–7-mm arcuate scleral tunnel incision was created 2 mm posterior to the superior limbus. A new foldable posterior chamber IOL was implanted, and its haptics were secured to the sclera at fixation points 3–4 mm posterior to the limbus using a double-armed 9-0 polypropylene suture with curved needles. The suture tension was adjusted to achieve stable centration of the IOL optic and alignment with the visual axis. The viscoelastic material was removed by irrigation and aspiration, and the scleral tunnel incision and conjunctival flap were closed with interrupted 10-0 nylon sutures. For patients undergoing combined anterior vitrectomy, a curved 23G needle was used to release adhesions between the lens capsule and the IOL. After safe extraction ofthe IOL, vitreous that entered the anterior chamber was resected, and no IOL or a new foldable posterior chamber IOL was implanted according to the situation. Finally, viscoelastic was removed, and the scleral incision was sutured intermittently with 10-0 nylon sutures. For patients undergoing combined posterior vitrectomy, conventional vitrectomy was performed using a three-port vitrectomy system. After completion of vitrectomy, the posteriorly dislocated IOL was carefully brought into the anterior chamber and extracted through the scleral tunnel incision.

### 2.3 Follow-up and evaluation

Preoperative and postoperative data and surgical records were retrospectively retrieved from the hospital medical record system. Collected data included gender, age, postoperative lens positional parameters (lens tilt and decentration degrees), preoperative comorbidities and postoperative complications, as well as UDVA, CDVA, IOP and ECD before surgery, at 1 week and 6 months postoperatively. Preoperative and postoperative UDVA and CDVA were recorded by Snellen charts and converted to logarithm of the minimum angle of resolution (logMAR) for statistical analysis; manual counting and manual movement were assigned values of 1.98 and 2.28 logMAR, respectively. Biometric parameters including AL, anterior chamber depth (ACD), lens thickness (LT) and white-to-white (WTW) were measured using IOL Master 700 (Carl Zeiss, Germany); IOP was measured using a non-contact tonometer (Canon TX-20, Tokyo, Japan); Two-dimensional (2D) and three-dimensional (3D) IOL tilt and decentration were measured using anterior segment optical coherence tomography (CASIA-2; Tomey Corporation, Nagoya, Japan). Two-dimensional IOL tilt was defined as the angular deviation of the IOL optical axis from the reference axis within an individual meridional cross-sectional image, whereas 2D decentration was defined as the linear displacement of the IOL center from the reference axis within the same plane (16). Three-dimensional IOL tilt and decentration were automatically derived from the spatial reconstruction of multiple radial scans and represented the resultant spatial tilt angle and decentration distance, respectively (17). ECD and central corneal thickness (CCT) were measured using a corneal endothelial microscope; SCP/DCP vessel density, retinal thickness of the operative and Control Eyes were measured using WF-OCTA. A regional measurement was considered technically invalid if the corresponding image showed at least one of the following quality defects: (1) evident motion artifacts resulting in vascular displacement, duplication, or discontinuity; or (2) substantial signal loss or uneven signal intensity preventing clear visualization of the vascular network.

### 2.4 Statistical analysis

All statistical analyses were performed using SPSS software. Continuous variables were expressed as the mean ± standard deviation, and categorical variables were presented as frequencies and percentages. Before analysis, normality was assessed using Q-Q plots and the Shapiro–Wilk test, and homogeneity of variance was evaluated where applicable. Longitudinal changes in visual acuity and intraocular pressure (IOP) were analysed using linear mixed-effects models, with time included as a fixed effect and patient included as a random intercept to account for the within-patient correlation among repeated measurements. Preoperative measurements were specified as the reference category. The normality and homoscedasticity of model residuals were assessed. Corneal endothelial cell density (ECD), which was assessed at two time points, was compared using a paired-samples t-test after confirming the approximate normality of the paired differences. When the assumptions for parametric analyses were not adequately satisfied, the corresponding nonparametric methods were used. IOL tilt and decentration magnitudes were compared using linear mixed-effects models, with group as a fixed effect and matched-pair identification as a random intercept. Orientation angles were summarized using the circular mean and circular standard deviation and compared using a paired circular permutation test that preserved the matched-pair structure. All tests were two-sided, and *P* < 0.05 was considered statistically significant, except for the exploratory OCTA analyses. The unadjusted *P* values for the OCTA comparisons were interpreted as descriptive rather than confirmatory. For the OCTA comparisons, linear mixed-effects models were fitted with group (operated versus control) as a fixed effect and matched-pair identification as a random intercept. Operated eyes were individually matched 1:1 with independent control eyes by age and sex.

## 3 Results

### 3.1 Baseline characteristics of patients

Table 1 summarizes the baseline clinical characteristics of the enrolled patients. A total of 28 patients (28 eyes) were included in this study, including 14 males and 14 females, with a mean age of 46.32 ± 8.27 years. Among the enrolled patients with high myopia, 18 cases (64.3%) had a history of previous retinal detachment- related surgery. The main chief complaint at admission was blurred vision or decreased visual acuity in 20 patients (71.4%), followed by monocular diplopia in 6 patients (21.4%), 1 patient (3.6%) was admitted due to pain after trauma, and 1 patient (3.6%) complained of photophobia.

**Table 1:** Demographics and clinical characteristics at baseline.

Parameters	Mean ± SD
Number of patients	28
Age（year）	46.32 ± 8.27
gender（female/male）	14/14
Eye Laterality（OS/OD）	13/15
Axial length（mm）	28.03 ± 3.46
White-to-white distance（mm）	11.86 ± 0.45
Time since the initial surgery（year）	7.14 ± 7.34
Number of IOL *in situ* repositioning	6
Number of IOL explantation without secondary IOL implantation	10
Number ofScleral fixation of IOL	12

OD, oculus dexter, OS, oculus sinister. IOL, intraocular lens.

The causes ofIOL dislocation included no obvious cause in 24 eyes (85.7%), trauma in 1 eye (3.6%), and occurrence after strenuous exercise in 3 eyes (10.7%).

### 3.2 Visual acuity outcomes

The preoperative and postoperative visual acuity, SE, and IOL are detailed in Table 2.The preoperative UDVA of patients was 0.89 ± 0.58 log MAR. The UDVA at 1 week postoperatively was 0.86 ± 0.43 log MAR, with no statistically significant difference compared with the preoperative value (*P* = 0.893); the UDVA improved to 0.59 ± 0.46 logMAR at 6 months postoperatively (*P* = 0.005). CDVA improved from 0.47 ± 0.57 log MAR preoperatively to 0.38 ± 0.59 log MAR at 6 months postoperatively, with a statistically significant difference (*P* = 0.031). At 6 months postoperatively, the mean UDVA and CDVA were 0.56 ± 0.43 and 0.34 ± 0.56 log MAR, respectively, in the IOL explantation without secondary IOL implantation group, with corresponding mean changes from baseline of −0.33 log MAR (95% CI, −0.71 to 0.05) and −0.13 log MAR (95% CI, −0.47 to 0.21), respectively. In the in-the-bag IOL repositioning group, the corresponding values were 0.50 ± 0.58 and 0.25 ± 0.69 log MAR, with mean changes from baseline of −0.28 log MAR (95% CI, −0.88 to 0.32) and −0.16 log MAR (95% CI, −0.65 to 0.33), respectively. In the sutured scleral fixation group, the corresponding values were 0.66 ± 0.45 and 0.47 ± 0.60 log MAR, with mean changes from baseline of −0.29 log MAR (95% CI, −0.63 to 0.05) and −0.03 log MAR (95% CI, −0.41 to 0.35), respectively. These subgroup results were descriptive and were not intended for inferential comparisons among the three surgical procedures.

**Table 2:** Refractive and IOP outcomes.

Parameters	Preoperative (Mean ± SD)	Postoperative (Mean ± SD)	Mean Change	95% CI	*P* Value
Postoperative Week 1 UDVA (logMAR)	0.89 ± 0.58	0.86 ± 0.43	−0.03	−0.48 to 0.42	0.893
Postoperative Week 1 CDVA (logMAR)	0.47 ± 0.57	0.45 ± 0.51	−0.02	−0.05 to 0.01	0.241
Postoperative 6 Month UDVA (logMAR)	0.89 ± 0.58	0.59 ± 0.46	−0.30	−0.50 to −0. 10	0.005**
Postoperative 6 Month CDVA (logMAR)	0.47 ± 0.57	0.38 ± 0.59	−0.09	−0. 17 to −0.01	0.031*
Spherical Equivalent, SE (D)	0.43 ± 1.20	−1.57 ± 2.83	−2.00	−4.22 to 0.22	0.075
Postoperative Week 1 IOP (mmHg)	16.78 ± 6.34	15.21 ± 3.53	−1.57	−3.70 to 0.56	0.143
Postoperative 6 Month IOP (mmHg)	16.78 ± 6.34	14.62 ± 4.74	−2. 16	−4.88 to 0.56	0.113
IOL explantation—6-month UDVA (logMAR)	0.89 ± 0.62	0.56 ± 0.43	−0.33	−0.71 to 0.05	-
IOL explantation—6-month CDVA (logMAR)	0.47 ± 0.60	0.34 ± 0.56	−0. 13	−0.47 to 0.21	-
IOL explantation—6-month IOP (mmHg)	17.20 ± 7. 10	14.18 ± 4.55	−3.02	−7. 10 to 1.06	-
In-the-bag IOL repositioning—6-month UDVA (logMAR)	0.78 ± 0.50	0.50 ± 0.58	−0.28	−0.88 to 0.32	-
In-the-bag IOL repositioning—6-month CDVA (logMAR)	0.41 ± 0.49	0.25 ± 0.69	−0.16	−0.65 to 0.33	-
In-the-bag IOL repositioning—6-month IOP (mmHg)	15.90 ± 4.80	14.68 ± 5.43	−1.22	−7.08 to 4.64	-
Sutured scleral fixation—6-month UDVA (logMAR)	0.95 ± 0.60	0.66 ± 0.45	−0.29	−0.63 to 0.05	-
Sutured scleral fixation—6-month CDVA (logMAR)	0.50 ± 0.60	0.47 ± 0.60	−0. 03	−0.41 to 0.35	-
Sutured scleral fixation—6-month IOP (mmHg)	16.87 ± 6.90	14.95 ± 4.95	−1.92	−5.93 to 2.09	-

CDVA, corrected distance visual acuity; UDVA, uncorrecteddistance visual acuity; IOP, intraocular pressure; SE,  spherical equivalent.

*P < 0.05,**P < 0.01, ***P < 0.001.

### 3.3 IOL positional outcomes

A comparison of postoperative IOL position between eyes after IOL dislocation surgery and eyes after routine cataract surgery is presented in Table 3. At 6 months postoperatively, the mean 2D IOL tilt and decentration in the operated eyes were 4.51 ± 2.17° and 0.45 ± 0.33 mm, respectively, while the mean 3D IOL tilt and decentration were 4.41 ± 2.09° and 0.44 ± 0.34 mm, respectively. The corresponding values in the post-cataract surgery control eyes were 4.06 ± 0.91°, 0.37 ± 0.12 mm, 3.58 ± 1.59°, and 0.43 ± 0.2 mm, respectively. No statistically significant between-group differences were observed in 2D IOL tilt (*P* = 0.434), 2D decentration (*P* = 0.347), 3D IOL tilt (*P* = 0.192), or 3D decentration (*P* = 0.962). Circular statistical analysis also showed no statistically significant differences in 3D tilt orientation (163.89 ± 100.88° vs. 125.44 ± 89.43°, *P* = 0.235) or 3D decentration orientation (172.72 ± 121.54° vs.132.22 ± 77.27°, *P* = 0.243) between the operated and control eyes.

**Table 3:** IOL position parameters after different surgeries (18 eyes).

Parameters	Post-IOL Dislocation Surgery	Post-Cataract Surgery	Mean Difference	95% CI	*P* Value
2D Tilt(°)	4.51 ± 2.17	4.06 ± 0.91	0.44	−0.71 to 1.59	0.434
2D Decent（mm）	0.45 ± 0.33	0.37 ± 0.12	0.07	−0.09 to 0.24	0.347
3D Tilt(°)	4.41 ± 2.09	3.58 ± 1.59	0.82	−0.43 to 2.08	0.192
3D Tilt @(°)	163.89 ± 100.88	125.44 ± 89.43	38.44	−26.13 to 103.02	0.235
3D Decent（mm）	0.44 ± 0.34	0.43 ± 0.2	0.01	−0.19 to 0.21	0.962
3D Decent @(°)	172.72 ± 121.54	132.22 ± 77.27	40.50	−28.95 to 109.95	0.243

Magnitude data are presented as mean ± SD and were compared using linear mixed-effects models. Orientation data are presented as circular mean ± circular SD and were compared using paired circular permutation tests.

*P < 0.05,**P < 0.01, ***P < 0.001.

## 4 Safety

### 4.1 Corneal endothelial function outcomes

Changes in corneal endothelial cell parameters before and after surgery data are shown in Table 4.The preoperative ECD of patients was 2672.72 ± 312.26 cells/mm^2^, which decreased to 2356.61 ± 420. 15 cells/mm^2^ at 6 months postoperatively, with a reduction of approximately 11.8% (*P* < 0.001); the average cell area (AVE) increased from 382.33 ± 60.80 μm^2^ to 443.27 ± 152.59 μm^2^ (*P* = 0.015). The coefficient of variation (CV) of corneal endothelial cells showed an increasing trend (from 36.33 ± 11.72% to 41.77 ± 11.78%, *P* = 0.073), and the percentage of hexagonal cells (HEX) showed a decreasing trend (from 48.22 ± 10.59% to 42. 16 ± 9.80%, *P* = 0.078); CCT showed an increasing trend (569.22 ± 40.2 μm preoperatively vs. 573.00 ± 39.46 μm postoperatively, *P* = 0.713). However, there were no significant differences in HEX, CV and CCT before and after surgery.

**Table 4:** Corneal endothelial function: preoperative vs. postoperative.

Parameters	Preoperative	Postoperative	*P* Value
CD	2672.72 ± 312.26	2356.61 ± 420.15	< 0.001***
CV	36.33 ± 11.72	41.77 ± 11.78	0.073
AVE	382.33 ± 60.80	443.27 ± 152.59	0.015*
PACH	569.22 ± 40.2	573.00 ± 39.46	0.713
HEX	48.22 ± 10.59	42.16 ± 9.80	0.078

CD, cell density, CV, coefficient of variation, AVE, average cell size, PACH, pachymetry, HEX, hexagonality.

*P < 0.05,**P < 0.01, ***P < 0.001.

### 4.2 Postoperative complications

DURING the 6-month follow-up, iris morphological changes occurred in 1 of 28 eyes (3.6%; exact 95% CI, 0.1%–18.3%), secondary glaucoma in 1 of 28 eyes (3.6%; exact 95% CI, 0.1%–18.3%), dry eye in 5 of 28 eyes (17.9%; exact 95% CI, 6.1%–36.9%), and cystoid macular edema in 1 of 28 eyes (3.6%; exact 95% CI, 0.1%–18.3%). No retinal detachment or bullous keratopathy occurred (0/28, 0%; exact 95% CI, 0%–12.3% for each). No IOL redislocation occurred among the 18 eyes with an IOL present postoperatively (0/18, 0%; exact 95% CI, 0%–18.5%).

### 4.3 Macular microcirculation and retinal thickness outcomes

The retinal thickness and vascular density in the operated eyes compared to the Control eyes are shown in Tables 5, 6, respectively. Across the nine anatomical regions, the operated eyes showed numerically greater mean retinal thickness in seven regions, greater mean SCP vessel density in seven regions, and greater mean DCP vessel density in seven regions than the matched control eyes.

**Table 5:** Retinal thickness across foveal, parafoveal, and perifoveal regions between operated eyes and control eyes (*N* = 28, region-specific sample sizes are indicated in the table where applicable).

Parameters	Foveal Region	Parafoveal Region				Perifoveal Region			
		Superior	Inferior	Temporal	Nasal	Superior	Inferior	Temporal	Nasal
Retinal Thickness (Operated Eye) (µm)	323.42 ± 95.92 （*N* = 27)	352.57 ± 60.38	346.26 ± 56.54	351 ± 57.07	355.15 ± 72.11	315.57 ± 32.79	294.21 ± 39.23	314 ± 76.31	323.47 ± 43.6
Retinal Thickness (Control Eye) (µm)	312.54 ± 65.34	358.45 ± 59.44 （*N* = 27)	324.78 ± 56.24	348.73 ± 55.24	336.42 ± 52.15	308.47 ± 39.62	290.26 ± 45.52	338.78 ± 59.74	312.7 ± 42.7
*p* Value	0.342	0.343	0.052	0.562	0.345	0.636	0.265	0.096	0.350

Regional retinal-thickness and vessel-density comparisons were exploratory, and the reported *P* values were used for descriptive purposes only.

**Table 6:** Retinal vascular density in foveal, parafoveal, and perifoveal subregions between operated eyes and control eyes (*N* = 28, region-specific sample sizes are indicated in the table where applicable).

Parameters	Foveal Region	Parafoveal Region				Perifoveal Region			
		Superior	Inferior	Temporal	Nasal	Superior	Inferior	Temporal	Nasal
Superficial Vascular Plexus (Operated Eye)	24.2 ± 13.23	35.7 ± 8.96	37.15 ± 9.87	35.23 ± 11.53 （*N* = 27)	33.24 ± 11.63	38.23 ± 7.34	37.37 ± 11.3	38.23 ± 7.33	39.51 ± 7.02
Superficial Vascular Plexus (Control Eye)	20.55 ± 11.37	32.9 ± 11.98	35.86 ± 10.34 (*N* = 27)	32.4 ± 10.53	32.52 ± 11.96	34.5 ± 10.52	38.02 ± 10.1	39.23 ± 8.29	35.45 ± 10.45
*p* Value	0.256	0.437	0.236	0.351	0.221	0.272	0.631	0.073	0.098
Deep Vascular Plexus (Operated Eye)	24.88 ± 12.73	36.32 ± 11.34	39.01 ± 8.39	39.99 ± 8.43	36.57 ± 9.03 （*N* = 27)	38.44 ± 8.53	39.52 ± 7.31	38.92 ± 6.98	38.29 ± 8. 1
Deep Vascular Plexus (Control Eye)	21.41 ± 10.45	34.21 ± 13.2 （*N* = 27)	38.42 ± 9.23	35.67 ± 8.35	35.32 ± 9.42	36.63 ± 9.32	38.3 ± 9.30	39.45 ± 9.07	38.91 ± 8.39
*p* Value	0.271	0.096	0.152	0.073	0.731	0.456	0.361	0.129	0.355

Regional retinal-thickness and vessel-density comparisons were exploratory, and the reported *P* values were used for descriptive purposes only.

### 4.4 Changes in IOP

The mean preoperative IOP was 16.78 ± 6.34 mmHg, decreasing to15.21 ± 3.53 mmHg at 1 week and 14.62 ± 4.74 mmHg at 6 months postoperatively. Neither postoperative value differed significantly from the preoperative value (*P* = 0.143 and *P* = 0.113, respectively). At 6 months postoperatively, the mean IOP was 14.18 ± 4.55 mmHg in the IOL explantation without secondary IOL implantation group, 14.68 ± 5.43 mmHg in the in-the-bag IOL repositioning group, and 14.95 ± 4.95 mmHg in the sutured scleral fixation group.

## 5 Discussion

This study systematically evaluated the clinical outcomes of different surgical approaches in terms of visual function recovery, IOL positional stability and ocular safety in patients with high myopia complicated by IOL dislocation. By comprehensively analyzing postoperative visual outcomes (UDVA, CDVA), IOL position (tilt and decentration) and safety (IOP, corneal endothelial changes, postoperative complications), this study aims to provide clinical evidence for surgical decision-making in patients with high myopia and IOL dislocation.

In terms of visual outcomes, different surgical treatments can achieve mid-to-long-term improvements in UDVA and CDVA in patients with high myopia and IOL dislocation. In contrast, there was no significant difference in UDVA at 1 week postoperatively compared with the preoperative value, which may be related to early postoperative corneal edema, anterior segment inflammatory reaction, surgically induced astigmatism fluctuation and ocular surface status (such as dry eye) (18). Although the mid-to-long-term postoperative CDVA was significantly higher than the preoperative value, the magnitude of improvement was lower than that reported in some previous studies on IOL dislocation surgery (19), which to a certain extent reflects the fundus structural limitations of high myopic eyes themselves. Patients with high myopia are often complicated with thinning of the retinal pigment epithelium and nerve fiber layer, parapapillary atrophy, and have a higher risk of retinal detachment (20–22). Among the patients with high myopia included in this study, 64.3% had a history of previous retinal detachment surgery, with varying degrees of damage to macular structure and retinal function, thus limiting the improvement of postoperative corrected visual acuity.

In this study, after treatment with different surgical approaches, the average IOL tilt was 4.51 ± 2.17° and the average decentration was 0.23 ± 0.33 mm in patients with high myopia and IOL dislocation. Compared with the previously reported sutureless scleral fixation techniques, these values were slightly higher than those of the Flanged Intrascleral Intraocular Lens Fixation with Double-Needle Technique proposed by Yamane et al. (average postoperative tilt of 3.4 ± 2.5°), and the 27 -gauge trocar-assisted sutureless intraocular lens fixation reported by Tatsuya Jujo et al. (average postoperative tilt of 3.52 ± 3.00°, average postoperative decentration of 0.39 ± 0.39 mm) (24, 25). It should be noted that unlike previous studies that mostly adopted a single surgical approach, the patients included in this study were mostly high myopic eyes with complex anatomical conditions, and some patients required combined vitrectomy to complete the surgery. Overall, the postoperative IOL tilt and decentration measurements were considered acceptable, among the 18 eyes retaining an IOL, 3 eye (16.7%) had a 2D IOL tilt ≥6°, whereas 2 eyes (2/18, 11.1%) had a 2D IOL decentration ≥0.6 mm (26, 27), indicating that the surgical approaches adopted in this study can achieve a stable and acceptable IOL position in high myopic eyes.

In this study, postoperative corneal ECD decreased from 2672.72 ± 312.26 cells/mm^2^ preoperatively to 2356.61 ± 420.15 cells/mm^2^ postoperatively (*P* < 0.001), with an overall reduction of approximately 11.8%. Although no clinical corneal decompensation was observed during follow-up, the significant reduction in ECD indicates measurable postoperative endothelial cell loss. Vaiciuliene et al. reported a 6.3% reduction in ECD after modified Siepser suture fixation (28), and Jo et al. reported a 1.5% reduction in patients who underwent repositioning of the original IOL or fixation of a new IOL in aphakic eyes (27). The possible reasons for the difference are that the surgical approaches for patients with high myopia and IOL dislocation in this study included combined vitrectomy and scleral suspension, which required more extensive disturbance of the anterior chamber and vitreous cavity, increasing the duration of intraocular manipulation and the risk of mechanical contact with corneal endothelium, thus resulting in endothelial cell loss; in contrast, the endothelial cell loss rate in this study was lower than that in patients with anterior chamber IOL (ACIOL) implantation reported by Vaiciuliene et al. (16.7% reduction, 2021 ± 480 cells/mm^2^ preoperatively vs. 1683 ± 446 cells/mm^2^ postoperatively) (27). This difference may be related to the persistent mechanical friction caused by the long-term contact between ACIOL and corneal endothelium in the anterior chamber. This study mainly adopted posterior chamber IOL repositioning or suspension surgery, which avoided long-term direct contact between IOL and corneal endothelium, and at the same time, every effort was made to protect endothelial cells from damage during IOL extraction and fixation.

In terms of IOP control, the overall postoperative IOP remained stable in this study with no significant fluctuation compared with the preoperative value, indicating good IOP safety in patients with high myopia and IOL dislocation in this study. During the follow-up period, the overall incidence of postoperative complications was low, and only a few mild and reversible complications were observed, including 1 case (3.6%) of CME and 1 case (3.6%) of iris morphological changes. Compared with other previously reported surgical approaches such as Yamane's sutureless scleral fixation, the incidence of intraocular hemorrhage was lower, which may be related to the intraoperative management of anterior/posterior vitreous and relief of vitreous traction, thus reducing the risk of perioperative hemorrhage (14, 28, 29). Notably, no severe complications such as intraoperative or postoperative retinal detachment and IOL redislocation were observed in this study, which further supports the overall safety and clinical controllability of implementing different surgical approaches based on full assessment of anatomical conditions.

Previous studies on IOL dislocation repositioning or fixation surgery mostly focused on visual acuity recovery, refractive status, IOP changes and postoperative complications (such as CME, hemorrhage, corneal edema) as outcome measures, while quantitative assessment of changes in macular microcirculation was relatively limited. As a non-invasive imaging technology, WF-OCTA can simultaneously assess the perfusion of superficial and deep capillaries, and reflect retinal structural changes combined with macular thickness. In this study, the operative eyes of patients with high myopia were generally comparable to the Control eyes in terms of SCP and DCP vessel density and retinal thickness parameters and its mechanism may be related to the reduction of unnecessary traction during surgery, the avoidance of persistent inflammatory reactions, and the combined vitrectomy adopted for high-risk vitreoretinal status (30–32).

This study has several limitations. First, it was an exploratory, single-center retrospective with a limited sample size. The three surgical approaches were selected according to ocular anatomy and capsular support, resulting in clinically heterogeneous subgroups and potential confounding by indication. The small subgroup sizes precluded reliable adjusted or inferential comparisons among the surgical procedures.

## 6 Conclusion

In summary, for patients with high myopia complicated by IOL dislocation, a high-risk group, the selection of different surgical approaches according to the patients’ ocular conditions can achieve stable reconstruction of the intraocular optical system and improve mid-to-long-term visual function, while IOP remained stable and no clinical corneal decompensation was observed despite significant postoperative endothelial cell loss. Although the improvement in corrected visual acuity was modest, possibly because of pre-existing fundus lesions in patients with high myopia, the incidence of severe complications was low during the six-month follow-up. Postoperative OCTA comparisons showed regional variations in macular microcirculation and retinal thickness, without a consistent pattern between the operated and matched control eyes. The above results suggest that the selection of surgical approaches based on ocular anatomical conditions and capsular status has important application value in the clinical management ofIOL dislocation in high myopic eyes. However, further verification of its long-term efficacy and safety in prospective studies with larger sample sizes and longer follow-up times is still needed.

## Data Availability

The raw data supporting the conclusions of this article will be made available by the authors, without undue reservation.
